# Metformin Induces Apoptosis and Ferroptosis of Ovarian Cancer Cells Under Energy Stress Conditions

**DOI:** 10.3390/cells14030213

**Published:** 2025-02-02

**Authors:** Yulun Wu, Ziying Zhang, Minhui Ren, Yao Chen, Jingying Zhang, Jiarui Li, Feng Gao, Yongli Bao, Yanxin Huang, Xiaoguang Yang, Zhenbo Song

**Affiliations:** 1 NMPA Key Laboratory for Quality Control of Cell and Gene Therapy Medicine Products, Northeast Normal University, Changchun 130024, China; 2 National Engineering Laboratory for Druggable Gene and Protein Screening, Northeast Normal University, Changchun 130117, China

**Keywords:** metformin, ovarian cancer, ferroptosis, apoptosis, NDUFB4

## Abstract

As ovarian cancer progresses, increased glucose use causes a glucose shortage in the tumor microenvironment. Therefore, it is crucial to find drugs that can effectively kill cancer cells in this energy stress setting. Here, we propose an effective therapeutic strategy that combines nutrient restriction with metformin to combat tumors. This study investigated the effects of metformin on ovarian cancer cells under energy stress conditions, mimicking the nutrient-deprived tumor microenvironment. We revealed that Metformin (10 mM) significantly reduced cell viability and proliferation under glucose deprivation conditions. Furthermore, it enhanced apoptosis and ferroptosis, as demonstrated by alterations in apoptotic protein expression and elevated levels of lipid reactive oxygen species (ROS), malondialdehyde (MDA), lipid peroxidation (LPO), and Fe^2+^. Transcriptional profiling revealed significant alterations in genes related to iron homeostasis and oxidative phosphorylation. Moreover, Metformin was found to induce mitochondrial dysfunction without affecting mitochondrial DNA or the expression of enzymes in the tricarboxylic acid (TCA) cycle, resulting in decreased ATP production and compromised activities of the respiratory chain complexes. The direct interaction between metformin and the NDUFB4 subunit in mitochondrial complex I was corroborated through the application of cellular thermal shift assay (CETSA) and drug affinity responsive target stability (DARTS) assays. In vivo, the combination of metformin and fasting cycles significantly inhibited SKOV3 cell-derived xenograft tumors in immunodeficient mice. Altogether, we have demonstrated that Metformin potentiates apoptosis and ferroptosis in ovarian cancer cells under energy stress conditions by targeting the NDUFB4 subunit of mitochondrial complex I, thus laying the groundwork for clinical testing. This study, though limited to cellular and animal levels, provides valuable insights into the therapeutic potential of metformin in ovarian cancer treatment.

## 1. Introduction

Ovarian cancer, often diagnosed at advanced stages, poses a significant therapeutic challenge due to its aggressive nature and poor prognosis [1,2]. Common targeted therapies for ovarian cancer include PARP inhibitors, which exploit the “synthetic lethality” mechanism to induce cell death by inhibiting DNA repair pathways [3,4]. Additionally, in the management of advanced high-risk ovarian cancer, anti-angiogenic therapies, exemplified by bevacizumab, have demonstrated substantial efficacy in both first-line and maintenance settings. These agents specifically target the vascular endothelial growth factor (VEGF) pathway, thereby disrupting the tumor’s blood supply [5]. However, traditional ovarian cancer therapies are plagued by substantial side effects such as myelosuppression, hypertension, and an elevated risk of bleeding.

The tumor microenvironment is characterized by a range of stressors, including nutrient deficiency, hypoxia, and acidosis, which significantly influence the metabolic adaptations of cancer cells. In such hostile conditions, tumor cells often rely on oxidative phosphorylation (OXPHOS) as their primary energy source despite the well-documented preference for glycolysis in many cancer types. The lack of sufficient nutrients forces cancer cells to adapt their metabolism to survive and proliferate. Recent studies have highlighted the critical role of mitochondrial function in this metabolic shift, suggesting that cancer cells can exploit OXPHOS to maintain energy production even when glucose is scarce [6,7,8]. By targeting the metabolic vulnerabilities that arise from nutrient deficiencies and the reliance on OXPHOS, novel strategies can be designed to inhibit tumor growth and improve patient outcomes [9,10].

Metformin, an antihyperglycemic agent, has been implicated in modulating cancer cell metabolism and exhibiting potential anti-neoplastic effects, particularly through its impact on mitochondrial function [11,12,13,14]. Recent structural biology insights revealed that metformin and related biguanides bound to and inhibited mitochondrial complex I activity, providing a molecular basis for their metabolic effects [15]. There are also studies that showed that metformin activated Adenosine 5′-monophosphate-activated protein kinase(AMPK) by binding to Presenilin Enhancer 2(PEN2), which inhibited v-ATPase and led to therapeutic benefits [16,17]. Furthermore, recent studies have further clarified the role of metformin in targeting cancer metabolism, highlighting its potential as a cancer therapeutic agent [18,19,20]. Notably, the cytotoxicity of metformin is significantly enhanced under low glucose conditions [21,22].

Recent studies have revealed metformin is capable of inducing ferroptosis [23,24,25,26]. Ferroptosis, a novel form of regulated cell death, has garnered attention as a potential therapeutic approach in cancer. By targeting the unique metabolic and redox vulnerabilities of cancer cells, ferroptosis induction offers a potential avenue to overcome resistance to conventional therapies and enhance antitumor efficacy [27,28]. Ferroptosis is sensitive to alterations in cellular metabolism, particularly mitochondrial dysfunction [29,30,31]. The susceptibility of ferroptosis is modulated by mitochondrial function [32,33]. Mitochondria regulates ferroptosis through the modulation of iron metabolism, lipid peroxidation, and ROS generation [34,35,36]. Building on these findings, the potential of metformin to induce ferroptosis under conditions of energy stress, as well as the underlying mechanisms, warrants further investigation.

In our research, we explored the impact of metformin on ovarian cancer cells under energy stress conditions. We observed metformin-triggered apoptosis and ferroptosis in these cells, which was accompanied by mitochondrial abnormalities. A mechanistic study suggested that it was facilitated through the interaction between metformin and NADH:Ubiquinone Oxidoreductase Subunit B4(NDUFB4) in respiratory chain complex I, leading to the inhibition of complex I activity and increased ROS level. Metformin’s ability to target multiple cell-death pathways endows it with the potential to significantly enhance the efficacy of ovarian cancer treatment. By effectively inhibiting the progression of ovarian cancer, it offers a promising therapeutic approach, especially under conditions of energy stress. This not only broadens our arsenal against ovarian cancer but also holds the promise of improving patient outcomes and survival rates.

## 2. Materials and Methods

### 2.1. Cell Lines

SKOV3 and A2780 cell lines are frequently utilized in ovarian cancer research due to their distinct characteristics. A2780 cells, established from an untreated ovarian endometroid adenocarcinoma tumor, thus serving as an ideal model for observing the effects of various treatments and testing drug potency. Prior to experimentation, the cell lines were rigorously authenticated and meticulously screened to ensure the absence of mycoplasma contamination. Both SKOV3 and A2780 cells were maintained in RPMI-1640 (Thermo Fisher Scientific, Waltham, MA, USA) medium supplemented with 10% FBS. For energy stress conditions, cells were cultured in glucose-free, glutamine-free, and pyruvate-free DMEM medium supplemented with 1 mM glucose and 10% dialyzed FBS. The treatment of cellular energy stress involves the replacement of the growth medium with energy stress medium, followed by the supplementation with metformin to assess their impact on cellular metabolism and viability.

### 2.2. BrdU Cell Proliferation Assay

In this study, BrdU incorporation was quantified using the BrdU Cell Proliferation ELISA kit (Cat. 11647229001, Roche, Basel, Switzerland). Cells were plated in 96-well plates at a concentration of 1 × 10^4^ cells per well. The culture medium was replaced with an energy stress medium containing metformin and supplemented with BrdU labeling solution. After incubation for 12 h, BrdU uptake was quantified according to the manufacturer’s protocols. Cell proliferation was assessed by measuring the absorbance at 450 nm utilizing a microplate reader.

### 2.3. MTT Colorimetric Assay

Cells were plated into 96-well plates at a density of 1 × 10^4^ cells per well. Each experimental group contained three replicate wells. Once the cells had adhered, the basal growth medium was replaced with an energy stress medium supplemented with metformin. The cells were cultured for 20 h. Subsequently, each well was treated with 20 μL of MTT solution (5 mg/mL in PBS) and subsequently incubated for 4 h. The medium was then aspirated, and DMSO was added. The absorbance was measured at 492 nm using a microplate reader. The cell viability was evaluated through the measurement of absorbance values.

### 2.4. Annexin V-FITC/PI Apoptosis Assay

Cells were initially seeded into 6-well plates and allowed to adhere. The culture medium was then replaced with energy stress medium containing 10 mM metformin. Apoptosis was assessed using the FITC Annexin V Apoptosis Detection Kit I (Cat. 556547, BD Biosciences, Franklin Lakes, NJ, USA). After centrifugation collection, cells were gently washed twice with cold phosphate-buffered saline, and then 1 × 10^5^ cells were transferred into a new tube for further analysis. Subsequently, FITC and PI were added, followed by incubation for 15 min in the dark. Each tube was supplemented with 400 µL buffer. The stained cells were analyzed by flow cytometry within one hour to ensure optimal fluorescence detection.

### 2.5. MDA, LPO, and Iron Content Detection

Cells were seeded into 6-well culture plates and permitted to adhere. Subsequently, the culture medium was replaced with energy stress medium with 10 mM metformin. The cells were maintained in culture for a period of 12 h. In the control group, an equal volume of PBS was added instead of metformin, ensuring that the experimental conditions were identical. Levels of LPO, MDA, and iron content were measured using specific assay kits (Cat. A106-1-1, Cat. A003-4-1, and Cat. A039-2-1, Nanjing Jiancheng Bioengineering Institute, Nanjing, Jiangsu Province, China) according to the manufacturer’s protocols. All measurements were adjusted relative to protein concentrations.

### 2.6. NADP^+^/NADPH and GSH/GSSG Ratio Measurement

The NADP^+^/NADPH and the GSH/GSSG ratio levels were evaluated using the NADP^+^/NADPH Assay Kit (Cat. S0179, Beyotime, Shanghai, China) and the GSH/GSSG Assay Kit (Cat. S0053, Beyotime, Shanghai, China) according to the manufacturer’s instructions. The values obtained were standardized based on the protein content of the respective samples.

### 2.7. RNA-Seq Analysis

SKOV3 cells were initially cultivated in 10 cm diameter cell culture dishes. Subsequently, the culture medium was replaced with energy stress medium supplemented with 10 mM metformin. After treatment, the cells were harvested, centrifuged, and lysed in TRIzol (Cat. 15596018CN, Invitrogen, Carlsbad, CA, USA) reagent. The collected samples were promptly immersed in liquid nitrogen and subsequently stored at −80 °C. These samples were then forwarded to Novogene (Beijing, China) for RNA sequencing and bioinformatics analysis. The RNA sequencing and pathway enrichment analysis methods employed in this study were conducted by Novogene. For RNA sequencing, the Illumina platform was utilized to sequence the mRNA of specific tissues or cells under certain conditions. The sequencing data were then aligned to the reference genome to comprehensively and rapidly analyze the sequence and abundance of mRNA, as well as the gene structure and newly generated transcripts. Differential expression analysis was performed in three main steps. First, the raw read counts were normalized to correct for sequencing depth. Second, a statistical model was applied to calculate the probability (*p*-value) of differential expression through hypothesis testing. Finally, multiple hypothesis testing correction was conducted to obtain the false discovery rate (FDR) values. For pathway enrichment analysis, the NovoMagic (https://magic.novogene.com/customer/main#/homeNew) cloud analysis platform was used. This platform integrates various bioinformatics analysis tools and visualization software, allowing for the identification of significant pathways through the KEGG database (Release 109.0, 1 January 2024). The analysis involved converting read counts to FPKM or TPM based on the read count table and gene length table, followed by the generation of various graphs, such as bar charts, to display the results of sample expression, GO/KEGG annotation, and enrichment.

### 2.8. Immunoblotting

Cells were first seeded into 6-well plates and permitted to be attached. Subsequently, the medium was changed to an energy stress medium supplemented with 10 mM metformin, and the cells were cultured for 12 h. After washing the cells with PBS, protein extraction was performed using RIPA Lysis Buffer. Subsequently, the protein sample was mixed with the loading buffer and then boiled at approximately 100 °C for 10 min. Protein samples were resolved by SDS-PAGE based on their molecular weight. The proteins were transferred from the gel to the PVDF membrane by applying an electric field. The membrane was blocked with 5% non-fat milk and incubated with primary antibodies at a specified dilution overnight at 4 °C. After washing, the membrane was incubated with HRP-conjugated secondary antibodies for 1 h. The protein–antibody complexes were detected using an enhanced chemiluminescence substrate and visualized with a chemiluminescence imaging system, with exposure times adjusted between 10 s and 1 min depending on the signal intensity. The relative expression levels of target proteins were quantified by densitometry, normalized to β-actin as the reference protein. The antibodies are as follows: Caspase 3 antibody (Cat. 19677-1-AP, 1:1000, Proteintech, Wuhan, Hubei Province, China), Caspase 9 antibody (Cat.10380-1-AP, 1:1000, Proteintech, Wuhan, Hubei Province, China), β-actin antibody (Cat. 81115-1-RR, 1:2000, Proteintech, Wuhan, Hubei Province, China), GPX4 antibody (Cat. 67763-1-Ig, 1:1000, Proteintech, Wuhan, Hubei Province, China), Bax antibody (Cat. 50599-2-Ig, 1:1000, Proteintech, Wuhan, Hubei Province, China), FSP1 antibody (Cat. 20886-1-AP, 1:1000, Proteintech, Wuhan, Hubei Province, China), NRF2 antibody (Cat. 16396-1-AP, 1:1000, Proteintech, Wuhan, Hubei Province, China), Bcl2 antibody (Cat. 68103-1-Ig, 1:1000, Proteintech, Wuhan, Hubei Province, China), IDH1 antibody (Cat. 12332-1-AP, 1:1000, Proteintech, Wuhan, Hubei Province, China), NDUFB4 antibody (Cat. 27931-1-AP, 1:1000, Proteintech, Wuhan, Hubei Province, China), PDH E1 Alpha antibody (Cat. 18068-1-AP, 1:1000, Proteintech, Wuhan, Hubei Province, China), ACSL4 antibody (Cat. 22401-1-AP, 1:1000, Proteintech, Wuhan, Hubei Province, China), NCOA4 (E8H8Z) antibody (Cat. 66849, 1:1000, Cell Signaling Technology, Danvers, MAUSA), Apaf1 antibody (Cat. 29022-1-AP, 1:1000, Proteintech, Wuhan, Hubei Province, China).

### 2.9. Transmission Electron Microscopy Assay

SKOV3 cells were initially cultivated in 10 cm diameter cell culture dishes; after the cells had adhered, the growth medium was replaced with energy stress medium containing 10mM metformin. Following trypsinization, the cells were harvested into centrifuge tubes and fixed overnight at 4 °C in 4% glutaraldehyde. Subsequently, the cells were treated with 1% osmium tetroxide for 1 h. They were then subjected to sequential dehydration using graded ethanol and acetone solutions, followed by embedding in epoxy resin. Ultrathin sections were prepared and subsequently stained with uranyl acetate in aqueous solution, followed by lead citrate staining. Cellular ultrastructure was subsequently examined using transmission electron microscopy.

### 2.10. mtDNA Copy Number Detection

Genomic DNA was isolated from the cells using the Mammalian Genomic DNA Extraction Kit (Cat. S0026, Beyotime, Shanghai, China). The relative mtDNA copy number was quantified by real-time PCR using primers specific for the ND1 gene, normalized to nuclear DNA content. ND1 forward primer: CCATCACCCTCTACATCACC, ND1 reversed primer: AGTTTGAGTTTGATGCTCACC; B2M forward primer: TGCTGTCTCCATGTTTGATGTATCT, B2M reversed primer: TCTCTGCTCCCCACCTCTAAGT). The expression levels of mtDNA were measured in triplicate using the SYBR Green fluorescence-based quantitative method. The mtDNA copy number was normalized using the comparative threshold cycle (2^−ΔΔCT^) method.

### 2.11. ROS Measurement

Cells were first seeded into 6-well plates and permitted to attach; after the cells had adhered, the growth medium was replaced with energy stress medium containing metformin. Cells were subjected to a 30-min incubation with 10 μM Reactive Oxygen Species Dihydroethidium (DHE) (Cat. C1300-2, Applygen Technologies, Beijing, China), at 37 °C following a PBS rinse. After the staining process was completed, the cells were promptly subjected to flow cytometric analysis within one hour to ensure the accuracy and reliability of the results.

### 2.12. Cell Mitochondrial Complex I, III, and V Activity Assay

SKOV3 cells were seeded into 10 cm cell culture dishes. Subsequently, the culture medium was replaced with an energy stress medium supplemented with 10 mM metformin. The cells were incubated for 12 h. The activities of mitochondrial complexes I, III, and V in cells were assessed using the Cell Mitochondrial Complex I, III, and V Activity Assay Kits (Cat. E-BC-K834-M, Cat. E-BC-K836-M, and Cat. E-BC-K838-M, Elabscience, Wuhan, Hubei Province, China), following the instructions provided by the manufacturer. The values obtained were standardized based on the protein content of the respective samples.

### 2.13. Measurement of ATP Level

Cells were seeded into culture plates. Once adhered, the growth medium was replaced with an energy stress medium supplemented with metformin. Intracellular ATP levels were assessed using the ATP Assay Kit (Cat. S0026, Beyotime, Shanghai, China) according to the manufacturer’s instructions. The cell supernatant was removed, and cells were lysed with the lysis buffer on ice. The lysate was collected, diluted appropriately, and mixed with the ATP detection solution. ATP concentrations were quantified using a fluorescent microplate reader by comparing the fluorescence intensity to a standard curve generated from known ATP concentration.

### 2.14. Cellular Thermal Shift Assay (CETSA)

Cells were seeded into 10 cm culture dishes and allowed to grow for 24 h. The cells were then rinsed with PBS and harvested. Cells were resuspended in RIPA buffer supplemented with a complete protease inhibitor cocktail. The lysates were centrifuged at 12,000× *g* for 15 min at 4 °C, and the supernatants were collected. These lysates were incubated with 100 mM metformin for 1 h. Subsequently, the lysates were divided into 50 µL aliquots and heated at 50, 55, 60, 65, 70, or 75 °C for 5 min. The heated lysates underwent centrifugation at a speed of 12,000× *g* for a duration of 15 min, maintained at a temperature of 4 °C. The supernatants obtained were then transferred into microcentrifuge tubes. They were subsequently subjected to immunoblot analysis.

### 2.15. Drug Affinity Responsive Target Stability (DARTS) Assay

We employed the DARTS assay to identify the protein targets of metformin. This label-free method is based on the principle that the stability of target proteins increases upon binding to small molecules, which protects them from proteolysis. First, proteins lysate from cells were prepared by cell lysis buffer (Beyotime, Cat. P0013). The supernatant was then incubated with metformin (100 mM) for 2 h. Following incubation, 0.02% pronase (Cat. 10165921001, Sigma-Aldrich, Louis, MO, USA) was added for 30 min to degrade unbound proteins. Finally, the identified proteins were validated using immunoblotting to confirm the interaction.

### 2.16. Tumor Xenograft Analysis

All animal experiments were approved by the Institutional Animal Care and Use Committee (NENU/IACUC, AP20231210, Approval date 10 December 2023) at Northeast Normal University, China. Female BALB/c nude mice were purchased from Beijing Vital River Laboratory Animal Technology (Beijing, China). The SKOV3 cell line-derived xenograft tumor animal model is chosen for ovarian cancer research because of its strong tumorigenicity. Originating from ovarian adenocarcinoma, SKOV3 cells can form tumors in immunocompromised mice, making this model an ideal platform for studying ovarian cancer therapies. These mice were subcutaneously inoculated with 5 × 10^6^ SKOV3 cells. One week later, the mice bearing SKOV3 tumors were randomly allocated into two groups: one maintained on a normal diet and the other subjected to fast. Each dietary condition was bifurcated into two groups: a control group and a metformin-treated (200 mg/kg/d, administered by intragastric injection) group (*n* = 4). In the fasting group, the mice were deprived of food from 18:00 until 10:00 the following morning. After 4 weeks, the xenografts were removed and weighed. The size of the xenografts was assessed using the following formula: V = 0.5 × L × W^2^, where V represents the volume, L is the length, and W is the width of the xenograft.

### 2.17. Statistical Analysis

Each experiment was performed a minimum of three times independently, and the resulting data were analyzed utilizing IBM SPSS and GraphPad Prism software (GraphPad Prism 7.0). The t-test was used to evaluate differences between two groups, whereas ANOVA was applied for comparisons involving three or more groups. Statistical significance was defined as *p* values below 0.05.

## 3. Results

### 3.1. Metformin Inhibits Cell Viability and Proliferation in Ovarian Cancer Cells Under Energy Stress Conditions

**Figure 1:**
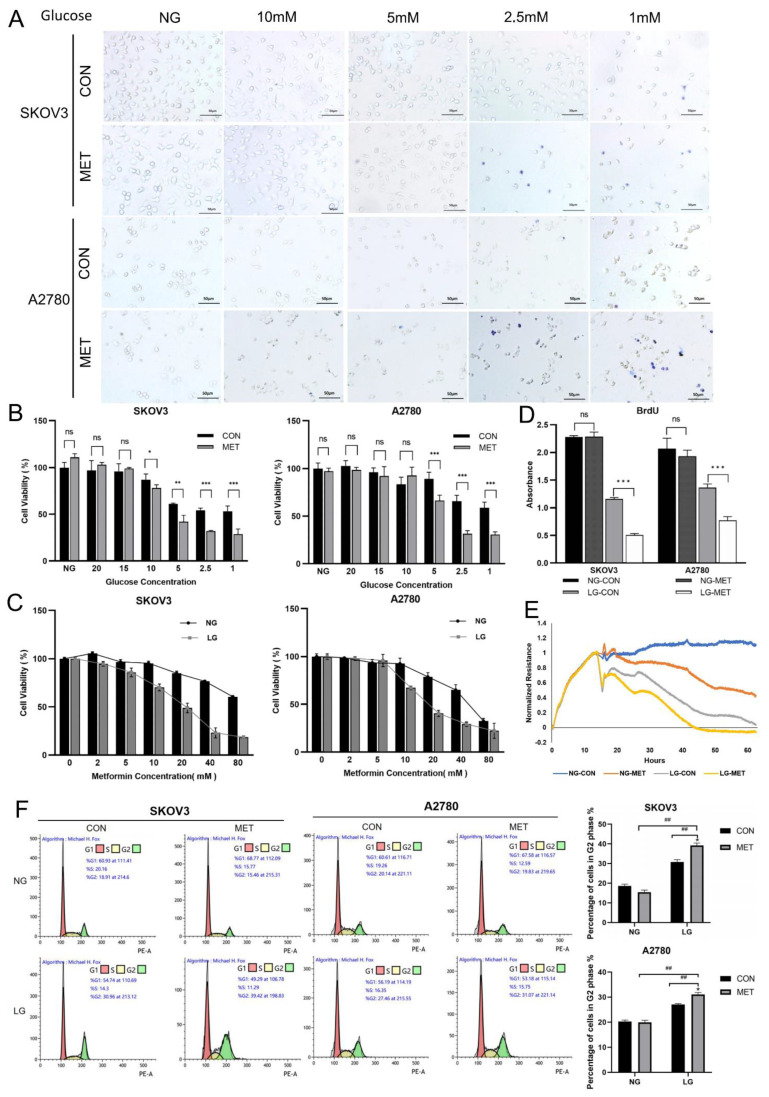
Metformin inhibits cell viability and proliferation in ovarian cancer cells under energy stress conditions. (**A**) Optical microscopy images depicting the Trypan Blue staining of SKOV3 and A2780 cells treated with 10 mM metformin in medium containing different concentrations of glucose. Normal glucose (NG) stands for RPMI1640 medium containing 25 mM glucose and 10% FBS, and others stand for DMEM medium without glutamine and pyruvate containing 10, 5, 2.5, 1 mM glucose, and 10% dialyzed FBS. (**B**) Cell viability was evaluated using the MTT assay following treatment with 10 mM metformin in media containing various glucose concentrations (normal glucose, 20, 15, 10, 5, 2.5, and 1mM). (**C**) Cell viability was assessed via the MTT assay following exposure to different concentrations of metformin (0, 2, 5, 10, 20, 40, and 80 mM) under normal glucose or low glucose conditions. The low glucose (LG) condition refers to DMEM medium that is devoid of glutamine and pyruvate, supplemented with 1 mM glucose and 10% dialyzed fetal bovine serum. (**D**) The BrdU absorbance of SKOV3 and A2780 cell lines was measured following a 12-h treatment with 10 mM metformin cultured in either NG or LG media. (**E**) The electrical impedance was measured in SKOV3 and A2780 cells following treatment with 10 mM metformin, cultured in either normal-glucose (NG) or low-glucose (LG) media for a duration of 12 h. (**F**) The cell cycle was analyzed using flow cytometry following a 12-h treatment with 10 mM metformin. Data were presented as mean ± SEM. The notation for statistical significance is as follows: * *p* < 0.05, **/## *p* < 0.01, *** *p* < 0.001, “ns” indicates “not significant” (*p* > 0.05). In figures where both symbols are present, */**/*** indicate significant difference between the groups in comparison to the NG-CON group, and ## indicate a significant difference between groups.

Initially, we examined the inhibitory effects of metformin on ovarian cancer cells cultured under varying glucose concentrations. Our study demonstrated that the introduction of metformin to low-glucose culture media, with glucose concentrations of 2.5 mM and 1 mM, resulted in a significant increase in the number of cells stained with trypan blue, indicating elevated cell mortality (Figure 1A). Conversely, under high-glucose culture conditions, metformin did not exhibit a significant impact on cell viability, and similar results were obtained when cell viability was examined (Figure 1B). Next, we conducted an analysis of the impact of varying concentrations of metformin on the viability of ovarian cancer cells. Our findings indicated that metformin exerted a dose-dependent inhibitory effect on these cells when exposed to low glucose conditions. (Figure 1C). Overall, the results showed that 10 mM metformin had no significant effect on cell viability under normal culture conditions. However, it significantly inhibited the viability of both cell lines under energy stress conditions. These findings led us to use 10 mM metformin as an effective concentration for studying its impact on ovarian cancer cells.

Furthermore, our data from BrdU incorporation and cell electrical impedance assays demonstrated that metformin markedly inhibited cell proliferation under conditions of energy stress (Figure 1D,E). This inhibitory effect was further corroborated by flow cytometry analysis, which indicated that metformin induced a G2 cell cycle arrest under the same stress conditions (Figure 1F). Taken together, our study indicated that both glucose limitation and metformin exposure synergistically reduced ovarian cancer proliferation and increased cell death under energy stress conditions.

### 3.2. Metformin Induces Apoptosis and Ferroptosis of Ovarian Cancer Cells Under Energy Stress Conditions

**Figure 2:**
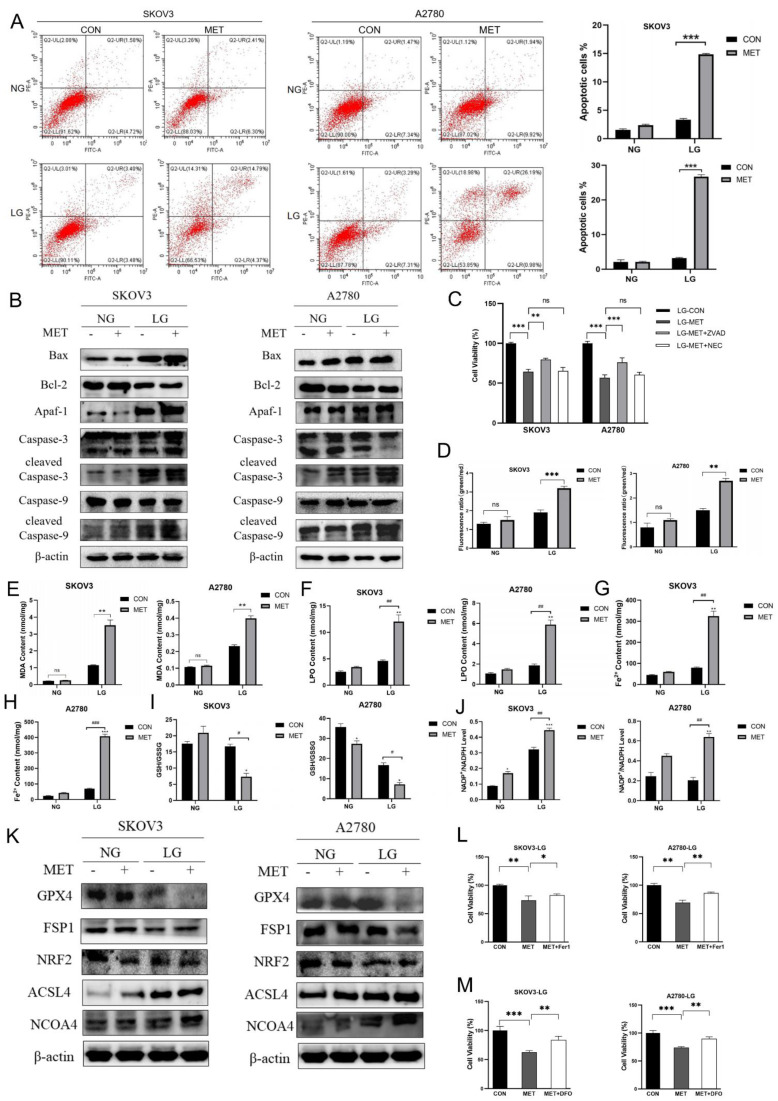
Metformin induces apoptosis and ferroptosis of ovarian cancer cells under energy stress conditions. (**A**) Cell death was assessed by Annexin V-FITC/PI staining after 12 h of treatment with 10 mM metformin. Cells were first gated based on forward scatter (FSC) and side scatter (SSC) to exclude debris and small particles. The cell population was then analyzed based on Annexin V-FITC and PI fluorescence. Quadrants were set to identify four cell populations: live cells (Annexin V-/PI-), early apoptotic cells (Annexin V+/PI-), late apoptotic cells (Annexin V+/PI+), and necrotic cells (Annexin V-/PI+). (**B**) Immunoblot analysis of apoptosis-associated proteins in SKOV3 and A2780 cells after 12 h of exposure to 10 mM metformin. (**C**) Cell viability was assessed using the MTT assay following a 12-h treatment with 10 mM metformin and 20 µM Z-VAD-FMK (ZVAD) or 100 µM Necrostatin-1 (NEC). (**D**) BODIPY581/591 staining was used to assess lipid ROS level. (**E**–**H**) Measurement of the MDA, LPO, and Fe^2+^ content. (**I**,**J**) Measurement of the GSH/GSSG and NADP+/NADPH ratios. (**K**) Immunoblot analysis of ferroptosis-related proteins after treatment with 10 mM metformin for 12 h. (**L**,**M**) Cell viability was assessed using the MTT assay following a 12-h treatment with 10 mM metformin and 10 µM ferrostatin-1 (Fer-1) or 100 µM Deferoxamine (DFO). Normal glucose (NG) stands for RPMI1640 medium containing 25 mM glucose and 10% FBS; low-glucose (LG) stands for DMEM medium without glutamine and pyruvate, containing 1 mM glucose and 10% dialyzed FBS; MET stands for the metformin group treated with 10 mM metformin; CON stands for the control group treated with PBS at an equal volume to that of metformin. Data were presented as mean ± SEM. */# *p* < 0.05, **/## *p* < 0.01, ***/### *p* < 0.001, “ns” indicates “not significant” (*p* > 0.05). In the diagram with both symbols, */**/*** indicate the significant difference between the groups compared to the NG-CON group, and #/##/### indicate a significant difference between groups.

In the subsequent investigation, we explored the effects of metformin on the apoptosis of ovarian cancer cells under energy stress conditions. Under energy stress conditions, treatment with metformin significantly elevated the apoptosis rates in SKOV3 and A2780 cells (Figure 2A). This effect was accompanied by notable alterations in the expression of proteins involved in apoptotic pathways. Specifically, the levels of Bcl-2 were reduced, whereas those of Bax were increased. Additionally, an enhanced cleavage of caspases 3 and 9 was observed, indicating the initiation of apoptotic cell death (Figure 2B). The addition of the apoptosis inhibitor Z-VAD-FMK significantly rescued the reduced cell viability induced by metformin, while the application of necroptosis inhibitor Necrostatin-1 failed to rescue the cells (Figure 2C). These results illustrated that metformin activated the mitochondria-dependent pathway to induce apoptosis in ovarian cancer cells. Abnormal mitochondrial function may lead to elevated mitochondrial ROS and subsequent ferroptosis. Therefore, we proceeded to investigate the impact of metformin on ferroptosis. The results indicated that metformin treatment under energy stress conditions resulted in an elevation in lipid ROS, MDA, LPO, and iron levels, along with a decrease in the GSH/GSSG ratio and an increase in the NADP^+^/NADPH ratio in SKOV3 and A2780 cells, suggesting the occurrence of ferroptosis (Figure 2D–J). Moreover, the expression of ferroptosis-suppressive proteins GPX4, FSP1, and NRF2 was decreased, while the expression of proteins ACSL4 and NCOA4 was increased after metformin treatment (Figure 2K). As expected, the addition of the ferroptosis inhibitor Ferrostatin-1 (Fer-1) and Deferoxamine (DFO) successfully rescued the reduced cell viability induced by metformin (Figure 2L,M). These findings demonstrated that metformin, under conditions of energy stress, not only induced apoptosis but also triggered ferroptosis.

### 3.3. Metformin Induces Transcriptional Changes in Ovarian Cancer Cells Under Energy Stress Conditions

**Figure 3:**
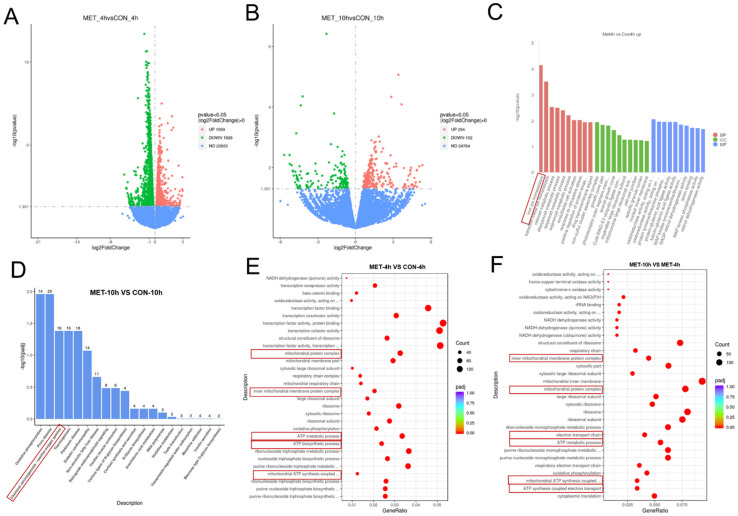
Metformin induces transcriptional changes in ovarian cancer cells under energy stress conditions. (**A**,**B**) Differential gene expression profiles of metformin-treated cells at 4 and 10 h compared to the control group. The volcano maps display the differential genes. (**C**) GO enrichment analysis histogram and the numerical values represent the number of differential genes enriched in this term. The distinct colors correspond to the three GO subclasses: Biological Process (BP), Cellular Component (CC), and Molecular Function (MF). (**D**) KEGG enrichment analysis scatter plot. (**E**,**F**) GO enrichment analysis histogram and the values represent the number of differential genes. Pathways related to mitochondrial complexes and ATP synthesis are highlighted within the red box. Normal glucose (NG) stands for RPMI1640 medium containing 25 mM glucose and 10% FBS; low glucose (LG) stands for DMEM medium without glutamine and pyruvate containing 1 mM glucose and 10% dialyzed FBS; MET stands for the metformin group treated with 10 mM metformin; CON stands for the control group treated with PBS at an equal volume to that of metformin.

We next investigated the mechanisms underlying metformin-induced cell death in ovarian cancer cells under energy stress conditions. The transcriptional profiles of SKOV3 cells were analyzed following metformin treatment. Our analysis revealed that 3488 genes were significantly altered at 4 h, with 446 genes showing significant changes at 10 h (Figure 3A,B). Among the genes that changed at 4 h, upregulated genes showed significant enrichment in iron homeostasis according to GO functional analysis (Figure 3C). KEGG (Kyoto Encyclopedia of Genes and Genomes) pathway enrichment analysis of the differentially expressed genes revealed that the ROS pathway was highlighted (Figure 3D). Additionally, GO (Gene Ontology) functional enrichment analysis further indicated significant changes in mitochondrial complex function and ATP metabolism across the 4-h and comparative 4-h versus 10-h time points (Figure 3E,F). These results suggested that metformin had a profound impact on cellular metabolism, stress response, and mitochondrial function under energy stress conditions, highlighting the critical role of metformin in regulating mitochondrial function under energy stress conditions and its subsequent effects on cell death.

### 3.4. Metformin Induces Mitochondrial Dysfunction in Ovarian Cancer Cells Under Energy Stress Conditions

**Figure 4:**
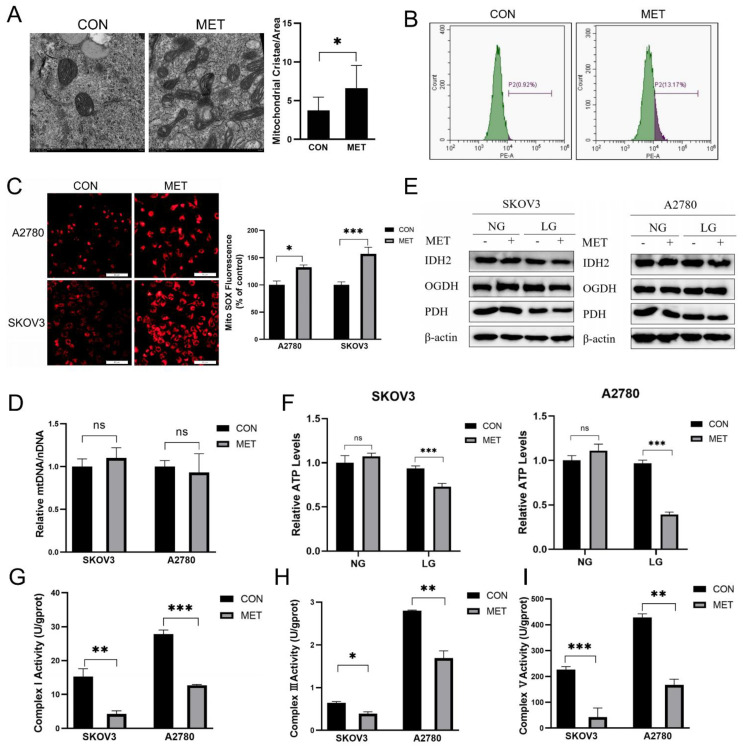
Metformin induces mitochondrial dysfunction in ovarian cancer cells under energy stress conditions. (**A**) The ultrastructure of mitochondria in SKOV3 cells was visualized using TEM, and number of mitochondrial cristae per mitochondrial area was analyzed (*n* > 5 mitochondria per group). (**B**) ROS levels were quantified using dihydroethidium (DHE) staining and analyzed by flow cytometry. (**C**) Mitochondrial superoxide levels were detected using the MitoSOX Red Mitochondrial Superoxide Indicator. (**D**) mtDNA copy number was evaluated in SKOV3 and A2780 cells following 12 h of treatment with 10 mM metformin. (**E**) Immunoblot analysis was performed to assess the expression of tricarboxylic acid (TCA) cycle enzymes in SKOV3 and A2780 cells after 12 h of exposure to 10 mM metformin. (**F**) Intracellular ATP levels were measured following 12 h of treatment with 10 mM metformin. (**G**–**I**) Mitochondrial Complex I, III, and V activity were detected. Normal glucose (NG) stands for RPMI1640 medium containing 25 mM glucose and 10% FBS; low glucose (LG) stands for DMEM medium without glutamine and pyruvate containing 1 mM glucose and 10% dialyzed FBS; MET stands for the metformin group treated with 10 mM metformin; CON stands for the control group treated with PBS at an equal volume to that of metformin. Data were presented as mean ± SEM. * *p* < 0.05, ** *p* < 0.01, *** *p* < 0.001, “ns” indicates “not significant” (*p* > 0.05).

Based on the sequencing data, which indicated alterations in mitochondrial function following metformin treatment, we conducted an examination of mitochondrial ultrastructure using transmission electron microscopy (TEM). Mitochondria exhibited alterations in cristae structure, indicative of mitochondrial dysfunction (Figure 4A). Concurrently, the elevation in ROS levels and mitochondrial superoxide were detected, further suggesting impaired mitochondrial function (Figure 4B,C). However, the mitochondrial DNA (mtDNA) copy number and expression of key enzymes in the TCA cycle remained unaltered, suggesting that the observed mitochondrial dysfunction was not due to changes in these fundamental components (Figure 4D,E). Consistently, the generation of ATP, a hallmark of mitochondrial function, was significantly reduced, and the activities of respiratory chain complexes I, III, and V were markedly decreased (Figure 4F–I). Collectively, these findings suggested that under conditions of energy stress, metformin treatment resulted in a marked impairment of mitochondrial function and cell death without affecting mtDNA or the expression of TCA cycle-related enzymes.

### 3.5. Metformin Targets NDUFB4 Subunit of Mitochondrial Complex I

**Figure 5:**
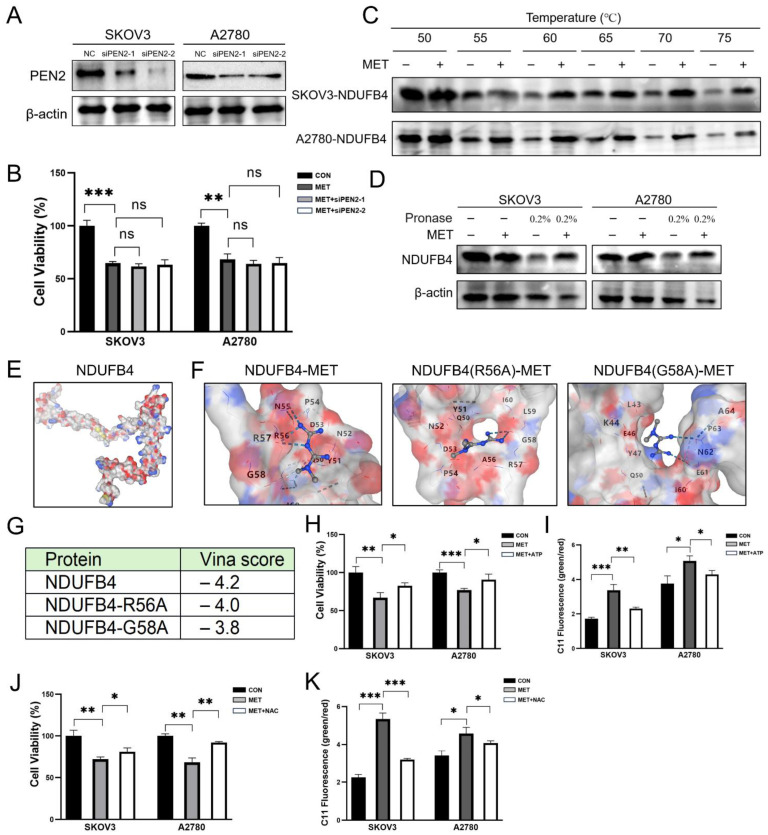
Metformin targets NDUFB4 subunit of mitochondrial complex I. (**A**,**B**) SKOV3 and A2780 cells were transfected with PEN2 siRNA (siPEN2-1 and siPEN2-2) and then treated with metformin. Cell viability was assessed using the MTT assay. (**C**) The effect of metformin on NDUFB4 protein stability in SKOV3 and A2780 cells was evaluated using the CETSA. (**D**) The impact of metformin on NDUFB4 protein stability was assessed by DARTS analysis. (**E**) The protein structure of NDUFB4 (UniProt: O95168) was predicted using AlphaFold 3. 3D structure of NDUFB4 displayed in surface representation, colored by element. Carbon atoms are shown in gray, hydrogen atoms in white, oxygen atoms in red, nitrogen atoms in blue. (**F**) Docking results of NDUFB4 protein, NDUFB4 R56A and G58A mutants with metformin. Molecular docking studies were performed using the AutoDock Vina software. Prior to docking, both the ligand and the receptor were prepared by removing any water molecules, adding hydrogen atoms, and performing energy minimization. (**G**) Vina scores representing the binding affinities of different docking results. (**H**) The rescue effect of ATP on metformin-induced cell death. (**I**) Lipid ROS was assessed by BODIPY581/591 staining after ATP treatment. (**J**) The rescue effect of NAC on metformin-induced cell death. (**K**) Lipid ROS was assessed by BODIPY581/591 staining after NAC treatment. Normal glucose (NG) stands for RPMI1640 medium containing 25 mM glucose and 10% FBS; low glucose (LG) stands for DMEM medium without glutamine and pyruvate containing 1 mM glucose and 10% dialyzed FBS; MET stands for the metformin group treated with 10 mM metformin; CON stands for the control group treated with PBS at an equal volume to that of metformin. Data were presented as mean ± SEM. * *p* < 0.05, ** *p* < 0.01, *** *p* < 0.001, “ns” indicates “not significant” (*p* > 0.05).

Previous studies have revealed metformin targeted PEN2, thereby activating AMPK and modulating mitochondrial metabolism and dynamics [16,17]. Recent advancements in structural biology have demonstrated that metformin and related biguanides bind to and inhibit complex I, specifically interacting with the NDUFB4 subunit [15]. We first investigated whether PEN2 was involved in metformin-induced cell death. The knockdown of PEN2 did not significantly impact the inhibitory effects of metformin under conditions of energy stress, implying that PEN2 may not serve as the principal mediator of metformin’s effects in this context (Figure 5A,B). Utilizing Cellular Thermal Shift Assay (CETSA), we observed an increase in the thermal stability of NDUFB4 protein following metformin treatment, indicating a direct interaction between metformin and NDUFB4 (Figure 5C). Moreover, the DARTS assay further confirmed this interaction by showing a decreased sensitivity of NDUFB4 to protease degradation upon metformin treatment (Figure 5D). Based on the sequence of NDUFB4 (UniProt: O95168), we utilized AlphaFold3 to predict the protein structure (Figure 5E). Molecular docking studies were performed to elucidate the binding affinity and interaction pattern of metformin with NDUFB4 using the AutoDock Vina software (1.2.0). The docking results revealed that metformin formed a stable complex with NDUFB4. This effect is primarily mediated by interactions with key residues R56 and G58 within the active pocket (Figure 5F,G). The orientation of metformin within the active site of NDUFB4 is depicted in Figure 5F, highlighting the key interactions that contribute to the binding affinity. These findings suggested that metformin impaired mitochondrial function through its interaction with the NDUFB4 subunit of mitochondrial complex I.

The inhibition of mitochondrial complexes will result in a reduction in ATP synthesis, as previously illustrated in Figure 4F. Consistent with this, supplementation of ATP partially rescued cell viability and reduced lipid ROS induced by metformin (Figure 5H,I). Additionally, the administration of the ROS scavenger N-Acetylcysteine (NAC) also significantly restored cell viability and mitigated lipid ROS (Figure 5J,K).

### 3.6. Metformin Inhibits Tumor Growth and Under Fasting Conditions

**Figure 6:**
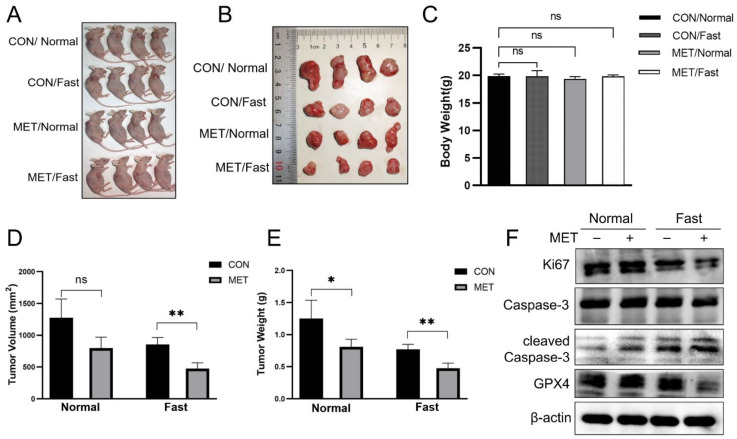
Metformin inhibits tumor growth and under fasting conditions. (**A**,**B**) Imaging of xenograft tumors in mice inoculated with SKOV3 cells after various treatments. CON/Normal stands for nude mice treated with normal feeding and saline intragastric administration; CON/Fast stands for nude mice treated with fasting feeding and saline intragastric administration; MET/Normal stands for nude mice treated with normal feeding and metformin intragastric administration; MET/Fast stands for mice treated with fasting feeding and metformin intragastric administration. (**C**) Body weights of mice in different experimental groups. (**D**,**E**) Tumor weight and volume. (**F**) Immunoblot analysis of Ki67, cleaved Caspase-3, and GPX4 in tumor-derived lysates. *n* = 4 biologically independent replicates. Data were presented as mean ± SEM. * *p* < 0.05, ** *p* < 0.01, “ns” indicates “not significant” (*p* > 0.05).

To elucidate the effects of metformin in vivo, we examined its influence on immunodeficient mice maintained under normal feeding and fasting conditions (*n* = 4 per group). SKOV3 cells were implanted subcutaneously into female nude mice. One week later, the mice were divided into four groups: two groups were allowed ad libitum feeding, while the other two groups underwent fasting. Each feeding condition included a control group and a metformin-treated group. Under normal feeding conditions, metformin did not significantly affect tumor growth. However, in the metformin-treated fasting group, both tumor volume and weight were significantly decreased compared to the control groups (Figure 6A,B,D,E). These results indicate that metformin’s tumor-inhibitory effects are potentiated under fasting conditions. Additionally, there were no significant differences in body weight among the different groups (Figure 6C). Moreover, we detected that the levels of Ki67 and GPX4 were decreased, whereas the level of cleaved Caspase3 was elevated in tumor tissues from the MET/Fast group, indicating weakened proliferation and increased apoptosis and ferroptosis (Figure 6F). These data indicated a potential synergistic effect of metformin and dietary restriction on tumor suppression.

## 4. Discussion

Our study has unveiled the significant role of metformin in inducing apoptosis and ferroptosis of ovarian cancer cells under energy stress conditions, highlighting a metabolic vulnerability that could be therapeutically targeted. In vivo studies conducted on immunodeficient mice reveal the potential synergistic effects of metformin combined with dietary restriction in tumor suppression, thereby highlighting the drug’s therapeutic promise beyond in vitro environments. Under energy stress conditions, metformin can trigger cell death, offering a potential therapeutic strategy that can be integrated with existing cancer treatments.

Previous research in ovarian cancer treatment has primarily focused on developing novel targeted drugs and exploring immunotherapy strategies. Many studies have aimed to design small-molecule inhibitors against specific oncogenes to block abnormal signaling pathways in cancer cells [3]. However, these inhibitors often encounter drug resistance, limiting their clinical application. Immunotherapy-based approaches, which aim to harness the immune system to attack cancer cells, are frequently restricted by the immunosuppressive tumor microenvironment of ovarian cancer [37,38]. In contrast, our current study presents several distinct advantages. Firstly, we have adopted a novel approach by simulating drug effects under conditions that closely mimic the tumor microenvironment, allowing for a more accurate reflection of the real-life growth environment of tumors and deeper insights into drug efficacy and resistance mechanisms. Secondly, we have chosen to investigate metformin, a drug already widely used in clinical practice with well-established safety and tolerability profiles [39,40]. Our findings demonstrate that metformin can effectively induce apoptosis and ferroptosis in ovarian cancer cells, opening up a new metabolic intervention pathway that could be combined with existing therapies to enhance treatment efficacy and reduce side effects.

Recent studies have demonstrated that although apoptosis and ferroptosis exhibit significant differences in mechanisms and morphological characteristics, they are closely interconnected. This interconnection is not only reflected in gene regulation and oxidative stress but may also be mediated through shared signaling pathways that facilitate their synergistic effects. A deeper investigation into the interplay between these two modes of cell death is of great significance for understanding disease mechanisms and developing novel therapeutic strategies [41,42]. The concurrent activation of apoptotic and ferroptotic cell death pathways may augment therapeutic efficacy and potentially circumvent resistance mechanisms, thereby offering a novel strategy for cancer treatment. Despite the challenges of inducing ferroptosis, such as the lack of specific biomarkers, potential off-target effects on immune cells, and complex crosstalk with other pathological processes, ferroptosis holds significant potential in cancer therapy by overcoming drug resistance, enhancing immunotherapy responses, and directly inducing tumor cell death.

Cancer cells often exhibit altered metabolic pathways to meet their increased energy demands. For instance, the Warburg effect, characterized by enhanced glycolysis even in the presence of oxygen, reflects a metabolic adaptation that allows cancer cells to thrive under nutrient-deprived conditions. However, recent studies have shown that functional mitochondria are essential for the survival and proliferation of many cancer types, suggesting that targeting mitochondrial metabolism could be a promising therapeutic strategy [43,44]. The therapeutic potential of targeting mitochondrial pathways has been explored in various contexts. For instance, the antibiotic mefloquine has been shown to impair mitochondrial function in cervical cancer cells, leading to apoptosis and reduced tumor growth [45]. Similarly, silencing the expression of VDAC1, a mitochondrial protein, has been found to induce metabolic rewiring, promoting differentiation and inhibiting tumor growth [46]. These findings underscore the importance of mitochondrial metabolism in cancer biology and suggest that strategies aimed at modulating mitochondrial function could enhance the efficacy of existing treatments and overcome therapeutic resistance.

Metformin has garnered significant attention for its potential role in cancer treatment, particularly due to its ability to target various metabolic pathways involved in tumorigenesis. Numerous studies have explored the antitumor mechanisms of metformin, highlighting its multifaceted actions that extend beyond glucose regulation. The divergence between studies identifying PEN2 as a metformin target and our findings implicating NDUFB4 may be attributed to the complex and multifaceted nature of metformin’s effects on cellular processes. The pleiotropic actions of metformin could result in differential targeting contingent upon the cellular context or the specific experimental conditions employed across various studies. Furthermore, the drug’s capacity to indirectly influence proteins via intricate interactions within cellular networks may account for the observed discrepancies. To reconcile these findings, it is imperative to undertake comprehensive studies that encompass multiple potential targets and their interactions across diverse conditions. Such investigations would ultimately yield a more nuanced understanding of metformin’s mechanism of action. The study on metformin-induced apoptosis and ferroptosis in ovarian cancer cells is limited by its reliance on in vitro and animal models, which may not fully reflect the clinical complexity. Future work should address these limitations by exploring the therapeutic potential in human cohorts.

Overall, the findings from these studies emphasize the multifaceted mechanisms by which metformin exerts its anticancer effects, particularly under conditions of energy stress. By targeting critical metabolic pathways and inducing cellular stress responses, metformin presents a compelling option for the treatment of ovarian cancer, warranting further investigation into its clinical applications.

## 5. Conclusions

Metformin significantly reduced ovarian cancer cell viability and proliferation under glucose deprivation conditions by enhancing apoptosis and ferroptosis. This effect was mediated by targeting the NDUFB4 subunit of mitochondrial complex I, leading to mitochondrial dysfunction and decreased ATP production. In vivo studies showed that combining metformin with fasting cycles effectively inhibited tumor growth in an SKOV3 xenograft mouse model. These results suggest that metformin has therapeutic potential for treating ovarian cancer through metabolic targeting. However, it is important to note that the study has limitations, such as a small sample size in the animal model, lack of validation in human primary cells, and the need for further clinical validation.

## Data Availability

Data are contained within the article.
